# The factors influencing the efficiency of drug-coated balloons

**DOI:** 10.3389/fcvm.2022.947776

**Published:** 2022-10-12

**Authors:** Zheng Cao, Jun Li, Zhao Fang, Yushanjiang Feierkaiti, Xiaoxin Zheng, Xuejun Jiang

**Affiliations:** ^1^Department of Cardiology, Renmin Hospital of Wuhan University, Wuhan, Hubei, China; ^2^Cardiovascular Research Institute, Wuhan University, Wuhan, Hubei, China; ^3^Hubei Key Laboratory of Cardiology, Wuhan, Hubei, China

**Keywords:** drug-coated balloon angioplasty, drug-eluting balloon, PCI – percutaneous coronary intervention (PCI), in-stent restenosis (ISR), *de novo* coronary artery diseases

## Abstract

The drug-coated balloon (DCB) is an emerging percutaneous coronary intervention (PCI) device that delivers drugs to diseased vessels to decrease the rate of vascular stenosis. Recent clinical studies have demonstrated that DCBs tend to have both good safety and efficacy profiles, leading to extended application indications in the clinic, including in-stent restenosis (ISR) for metal stents such as drug-eluting stents (DESs), small vascular disease, bifurcation disease, large vascular disease, acute coronary syndrome (ACS), and high bleeding risk. However, some previous clinical data have suggested that DCBs performed less effectively than DESs. No studies or reviews have systematically discussed the improvement strategies for better DCB performance until now. Drug loss during the process of delivery to the target lesion and inefficient delivery of the coating drug to the diseased vascular wall are two key mechanisms that weaken the efficiency of DCBs. This review is the first to summarize the key influencing factors of DCB efficiency in terms of balloon structure and principles, and then it analyzes how these factors cause outcomes in practice based on current clinical trial studies of DCBs in the treatment of different types of lesions. We also provide some recommendations for improving DCBs to contribute to better DCB performance by improving the design of DCBs and combining other factors in clinical practice.

## Introduction

Currently, the rapidly developing technology of percutaneous coronary intervention (PCI) has alleviated pain and saved a large number of patients with coronary heart disease (CHD), greatly reducing the risk and cost of thoracotomy treatment. Drug-eluting stents (DESs) have been introduced to effectively cure *de novo* CHD by mechanical support and sustained release of antiproliferative drugs, and they still dominate the current interventional treatment of CHD. However, with increasing cases of PCI, a persistent concern is late thrombotic events (1), even with second-generation DES. The drug-coated balloon (DCB) is a half compliance balloon catheter technique with no metal implants compared with DES. The coating on the surface of the balloon consists of an excipient and drugs that inhibit intimal hyperplasia. When the balloon expands, the coating drug can be evenly delivered to the surface of vessels and rapidly absorbed by the intima, with a lasting impact on the vascular intima. This process may preserve the original anatomical integrity of the artery (2). DCBs have previously been used to treat stent restenosis and have been shown to be effective and safe, while other new indications are emerging, such as for bifurcation lesions, small vessel lesions, and high-risk bleeding (3–6).

However, in the PCI process, even if the balloon reaches the diseased vessel and successfully expands, it does not mean that the therapeutic effect will be satisfactory. The concentration of the drug on the vascular wall is the fundamental factor affecting the therapeutic effect. This article is the first to review the key factors affecting DCB efficiency and offer corresponding proposed solutions to improve DCB performance.

## Mechanisms of drug-coated balloons

**FIGURE 1:**
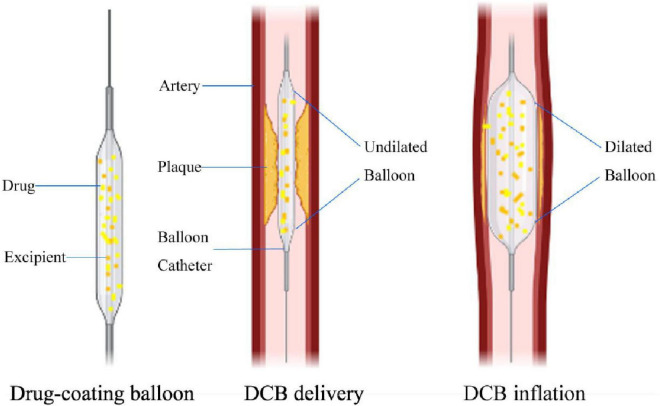
Mechanisms of DCB in longitudinal section view. The difference between DCB and plain old balloon angioplasty (POBA) is that the balloon surface is coated with antiproliferative drugs and excipients. When the DCB reaches the lesion, as the balloon expands, the antiproliferative drug on the balloon surface can be transferred to the vessel wall, and the average expansion time is 30 –60 s.

The mechanisms of DCBs are shown in Figure 1. The rationale for using DCBs derives from the notion that lipophilic drugs, such as paclitaxel, could be delivered to the vessel wall even with a short balloon inflation time. DCBs inhibit ISR using drugs that inhibit neointimal proliferation without the implantation of an exogenous device (7). Drugs are coated on balloons using excipients as drug carriers to facilitate adherence and release drugs during balloon inflation (8). The balloon delivery system can sustain a higher level of drug dose locally than through systemic delivery (9). To improve the efficiency of drug administration and effectively inhibit intimal hyperplasia, attention should be given to the lipophilicity of drugs, administration time, minimizing of drug loss in the process of transporting drugs to designated sites, protecting of the soluble drugs from blood erosion as much as possible, and strengthening of the binding ability of drugs (10).

## The influencing factors on drug-coated balloon efficiency

The efficiency of DCB delivery relies on various factors, namely, the type of coating drugs, effective excipients, the rate of pharmacokinetics, the optimal drug load, release kinetic profiles, and drug loss during the process of delivery (11, 12). In addition, proper lesion preparation and other related factors are also crucial to improving the therapeutic effects of DCBs.

### Influence of the type, dose, and chemical properties of the coating drugs on drug-coated balloon efficiency

A wide variety of different DCBs are available for PCI, and paclitaxel (PTX) remains one of the most preferred coating drugs, with a typical dose between 2 and 3.5 μg/mm^2^ (3). PTX inhibits cell proliferation by promoting the formation of microtubules and inhibiting the decomposition of microtubules during mitosis, thus stopping cells in the G2/M phase (13). Mohammed M proved that DCB-PEA (percutaneous endovascular angioplasty) could significantly reduce the activity of inflammatory proteases and postpone the progression of the disease, while the anti-inflammatory effect of PTX could shorten the double antiplatelet (DAPT) time in patients treated receiving DCBs (14). To increase the drug concentrations on the vascular walls, high crystalline and low soluble coatings were applied to the first generation of DCBs with large drug particles. The more particles that were deposited on the vasculature, the longer the effects lasted. However, using a highly crystalline coating could increase the toxicity of the organism and the possibility of particle embolism. Reducing the drug dosage could reduce concerns about particle embolization (15). *In vitro* tests of PTX showed that the proliferation of smooth muscle cells (SMCs) could be effectively inhibited as long as the IC50 of PTX was 1–2 ng/g, and the migration of SMCs could be inhibited when the IC50 of PTX was 0.4 ng/g (16–18). This level is sufficient to demonstrate that PTX can inhibit cell migration and proliferation at a low concentration. Interestingly, in a meta-analysis of mortality in patients treated with paclitaxel drug balloons at different drug doses, Peter A. Schneider pointed out that there was no statistically significant difference in mortality, especially for limb ischemia after DCB treatment with different doses of PTX (19), which could be due to the lipophilicity and tissue retention characteristics. PTX uptake kinetics are governed by the specific binding capacity to the arterial wall and are fairly independent of surface concentration. Even low drug densities might be associated with high local and potentially dangerous concentrations (20). PTX uptake does not obey concentration gradients, in characteristic contrast to hydrophilic drugs (21). This fact could be a reasonable explanation for the results of the above study. The potential mortality risk of paclitaxel has been controversial, and in a 2018 meta-analysis, Kantasanos suggested that paclitaxel drug balloons, as well as paclitaxel-eluting stents, had higher 2-year and 5-year mortality rates in patients treated for occlusive popliteal artery disease than in those treated receiving plain balloon angioplasty or bare-metal stents (22). Some scholars believe that the crystallization of PAC paclitaxel caused downstream embolisms (23), while others believe that the patients were treated with suboptimal secondary prevention medication (19). In his article, Dr. J. Nordanstig noted that there was little difference in mortality between the paclitaxel-coated balloon treatment group and the uncoated endovascular device treatment group during the 1- to 4-year follow-up period and suggested that the previously reported mortality might have been caused by bias in the meta-analysis (24).

In contrast, sirolimus balloon therapy has attracted increasing attention. Wim Martinet showed in his study that sirolimus alleviates atherosclerotic plaque formation by inhibiting macrophage proliferation, lipid accumulation, and plaque formation during angiogenesis (25). However, the systematic use of rapamycin in response to a number of adverse events, such as hypertriglyceridemia and interstitial lung diseases, and the topical use of rapamycin can reduce the incidence of adverse reactions (26), so rapamycin-coated balloons seem to be a proper choice. In previous studies of sirolimus stents, sirolimus prevented ISR by inhibiting smooth muscle progenitor cells and circulating progenitor cells, which could increase cardiovascular risk, while rapamycin-eluting stents tended to result in late thrombosis (27–29). Dr. Yvonne Patricia Clever applied sirolimus-coated balloons (SCBs) to the coronary arteries of pigs and confirmed that SCBs could effectively inhibit intimal hyperplasia, but the relevant clinical effect is uncertain (30). Combined with clinical trials of marketed approved SCBs (31, 32), in terms of major adverse cardiac events (MACEs) and target lesion revascularization (TLR), SCBs had the same effect as paclitaxel-coated balloons (PCBs) in the treatment of ISR in the short term, but SCBs must be confirmed by more clinical trials.

**FIGURE 2:**
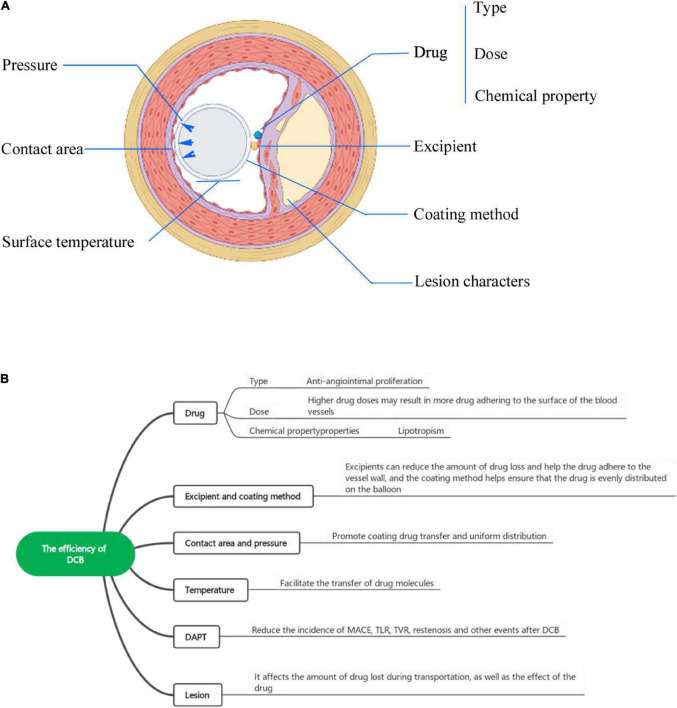
**(A)** The factors influencing DCB efficiency from a cross-sectional view. **(B)** The factors influencing the efficiency of DCBs include the drug (type, dose, chemical properties), excipient, coating method, balloon surface temperature, and lesion characteristics.

In addition to these two drugs, some other drugs have been used as coatings on drug capsules of implanted devices. In a report, arsenic trioxide was used to inhibit the growth and cycle of cancer cells and to induce the apoptosis of VSMCs *in vitro*. Scaffolds coated with arsenic trioxide effectively inhibited intimal hyperplasia in the iliac arteries of rabbits (33). This technology deserves to be extended to applications in DCBs, in addition to DESs. As analogs of sirolimus, the combination of adjuvant tamoxifen and immune molecule tacrolimus binding protein 12 (FKBP-12) has a high affinity with a strong inhibition of human coronary artery SMC proliferation. At the same time, compared with sirolimus, the adjuvant tamoxifen department has a short half-life in the body, and it has been proven to have weaker immune inhibition compared with sirolimus (34), reducing systemic exposure and side effects. Compared with PTX, zotarolimus is more lipophilic but less absorbed in the vascular wall (35). Studies have shown that zotarolimus-coated balloons (ZCBs) not only inhibit coronary artery inflammation but also inhibit the formation of new vascular neointima to a similar extent as DES (36). Clinical trials of zotarolimus-eluting stents in recent years have been relatively satisfactory in inhibiting intima proliferation and atherosclerotic plaque inflammation (37–39), but clinical trials comparing ZCB with other types of zotarolimus-coated stents have been lacking. Additionally, zotarolimus is less sensitive to complex lesions than paclitaxel (10, 40). Currently, the safety and efficacy of the above drug-related DES have been fully verified with a large amount of clinical data, but the corresponding DCBs remain insufficient. More clinical trials are necessary (Figure 2).

### Influence of excipients and coating methods on drug-coated balloon efficiency

Currently, lipophilic antiproliferation drugs are more suitable for coating, but lipophilic drugs have poor solubility. To decrease the loss of drugs caused by blood scouring and improve the stability of drug release, various carrier excipients are needed. The excipients currently used are hydrophilic excipients that can hydrate and gradually promote the release and transfer of the drug to the vessel wall. Early on, Dr. Bruno Scheller added PTX to an iopromide contrast agent and found that PTX in the contrast agent was several times more soluble than in normal saline and inhibited ISR without systemic or local toxicity (41, 42). All studies have shown that, in addition to iopromide, lemon acid butyryl three hydroxyl ester (BTHC) could also be used as an excipient of paclitaxel for drug delivery and organization reserve (43–45).

However, it is worth noting that the combination of lipophilic drugs and hydrophilic excipients will produce a highly crystalline coating, which is unstable and will form particles while the crystallization resolves, thus causing downstream vascular embolisms (31, 46). It is encouraging that changing the ratio of drug to excipient and improving the spraying method to maintain the stability of the coating could be effective methods to reduce the incidence of these events. Various experiments have shown that changing the ratio of drug to excipient actually changes the solubility, causing less crystalline coating formation and leading to less microparticle formation (47). Dr. Sebastian Kaule pointed out that a thin coating with a smooth surface and high delayed solubility could decrease drug scouring, as well as particle loading, which could further reduce embolization (48). Various methods, namely, impregnation, air spraying, and ultrasonic spraying, can be adopted to keep the surface of the DCB smooth to reduce the occurrence of embolization events caused by coating particles. Impregnation is one of the earliest methods of hydrophilic coating. However, with the increase in impregnation, the uniformity of the coating will become less ideal, and the deposition at the fold will also be affected by solution viscosity and drug concentrations. Although air spraying can produce a highly uniform coating, the drug transfer efficiency is still low (48, 49). Therefore, developing a new coating method is necessary, which not only can improve the drug uniformity on the coating but also can decrease the incidence of downstream vascular embolism events (Figure 2).

### Influence of the contact area and pressure between the drug-coated balloon and vascular wall on drug-coated balloon drug delivery

**FIGURE 3:**
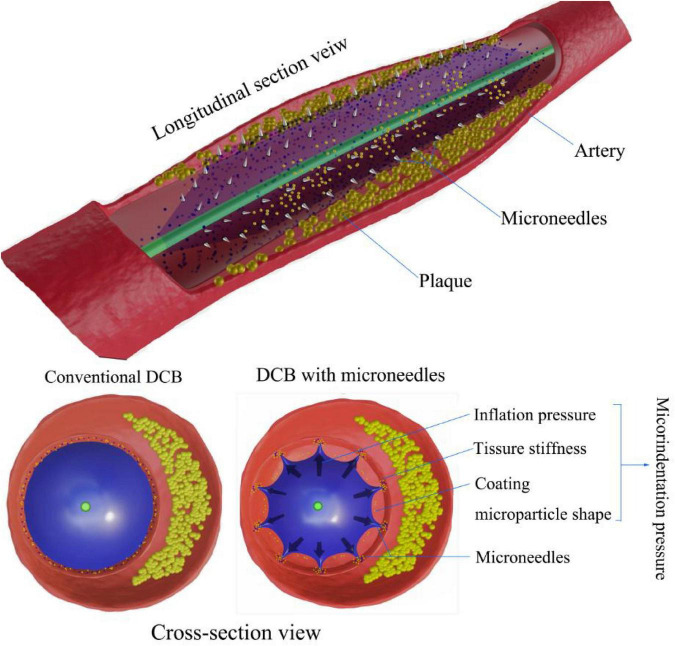
Microindentation pressure on DCB drug delivery. Compared with conventional DCBs, microindentation pressure results from the combination of inflation pressure, coating particle shape, and tissue stiffness. Microneedles of DCBs enhance drug delivery by increasing contact pressure in micromode after full inflation.

Figure 2 shows the factors influencing the contact area and microindentation pressure between the DCB and vascular wall. The contact area and pressure between the DCB and vascular wall also play important roles in drug delivery and retention in tissues and the efficiency of DCBs, but this relationship has been poorly studied until now. Abraham R. Tzafriri’s team developed a concept called microindentation pressure from the contact pressure gradient of the coating. Microindentation pressure is positively correlated with coating adhesion and van der Waals adhesion, which in turn promotes drug transfer. The factors influencing the microindentation pressure are shown in Figure 3 (50). Dr. Nicola Stolzenburg explored the effect of inflation pressure on drug delivery from DCBs to the vascular wall in an animal study, which demonstrated that higher inflation pressure could promote the metastasis of PTX in atherosclerosis (51). This finding could be explained by the contact pressure of the DCB on the atherosclerotic vessels perhaps being greater than that on the normal arteries. The coating adhesion, which is proportional to the microindentation pressure and van der Waals adhesion, promotes drug transfer (52).

In actual clinical interventions, a more defined range of expansion pressure should be considered. During interventional procedures, high inflating pressure not only can cause plaque rupture, aggravate the vascular injury, increase the possibility of vascular dissection, and even cause negative vascular remodeling and neointimal hyperplasia, but it also can lead to restenosis after angioplasty (53). In 2020, a team at Yonsei University designed a new type of DCB. Based on a rabbit model of atherosclerosis, the application of microneedles (MNs) on the balloon surface could enhance drug delivery efficiency as a form of increased intravascular drug dosing. Microneedles seem to be an emerging innovation in DCBs and related areas of intervention (54–57). The pressure of microindentation can be increased when the pressure of balloon inflation remains continuous (Figure 3). Regarding safety, compared with conventional balloons without drugs, MN balloons did not induce a greater intravascular immune response (50, 58). In addition to pressure, the contact area is another important factor that affects drug delivery. The inflated balloon is in contact with the extensive surface of the vessel, providing uniform longitudinal and lateral drug delivery to the intima and enabling uniform drug transfer and distribution along the length of the lesion (35). With the increase in the contact area between the coating and the vascular tissues, the adhesion of the coating will also increase, thus promoting the effective delivery of drugs (50, 51), which should be considered when designing new DCBs.

### Influence of the surface temperature of the drug-coated balloon on drug delivery

While the coating drug is retained in the arterial wall, diffusion and convective forces, generated by random molecules and solvent-driven flow, transfer the drug molecules deeper into the vessel wall (10), and then the drug molecules bind to the reversible extracellular matrix binding sites. This transmural transport is enslaved to the tissue binding ability, dissolution reaction, effective diffusivity, etc. (20). Recent studies have shown that temperature could be considered another important factor. Temperature not only changes the solubility of lipophilic antiproliferative drugs and thus affects drug release, but it also promotes the transfer of drug molecules into the deeper vasculature (59, 60). Current studies of temperature have been limited to the radiofrequency ablation of atherosclerotic plaques using balloon temperature, such as the PLOSA balloon catheter, which melts plaque by transferring heat to the vascular wall (61–63). However, the human body temperature is constant, and high temperatures could cause injury to the vessel endothelium, leading to intracoronary thrombosis and periarterial myocardial necrosis. Inflammation can lead to restenosis, which is another non-negligible challenge when using temperature to accelerate drug delivery in the process during radiofrequency ablation (64–66). Few studies have focused on the influence of temperature on the delivery efficiency of DCBs. More studies in this field are needed to promote the updating of DCB technologies in future.

### The coronary artery lesion characteristics on the efficiency of drug-coated balloon

The coronary anatomic factors affecting interventional surgery include the presence of calcium, severe vascular distortion, thrombotic content, and diffuse arteriosclerosis (67), which could also affect the drug delivery efficiency of DCBs. Dr. Karthic Anbalakan pointed out that the overall morphology and volume of atherosclerosis were important factors that affected the efficiency of DCBs (68). Fernandez reported a significant 3.5-fold increase in PTX levels in atherosclerotic lesions compared with control lesions (69).

In previous studies, it has been proposed that different compositions of atherosclerotic plaques have discrepant effects on the absorption and retention of drugs, the biological response after intimal injury, and the intimal pharmacological response to drugs (40, 70–72). Can the lipid components in atherosclerotic plaques affect the delivery of lipophilic drugs to arteries, such as paclitaxel and sirolimus? Studies have demonstrated that the deposition of these drugs in the human aorta is inversely proportional to the lipid content (40, 70). The lipid content in atherosclerotic plaques weakens the effectiveness of lipophilic drugs. In addition, DCBs are less effective in treating severely calcified blood vessels (40, 50), as proven in previous studies. Dr. F Fanelli found that DCBs were not effective in lesions with severe calcification, and corresponding tests proposed that the presence of calcium inhibited drug absorption (73). In addition, the extracellular matrix plays a key role in the distribution and retention of lipophilic drugs (40, 70). Because drugs bind to histone proteins for transmembrane transportation, these proteins can promote drug delivery and retention. Moreover, previous studies have indicated that vascular beds with more elastin have greater absorption and deposition capacity (10, 74). Lipophilic drugs preferentially bind to elastin and tend to be distributed in deeper layers of the arterial wall. Calcium ions can affect the structure of elastin and thus affect drug absorption, which requires more attention.

### Effect of preoperative lesion preparation on the efficacy of drug-coated balloon

Lesion preparation is very important for both drug-eluting stents and drug balloon implantation. The importance of lesion preparation for drug balloon implantation is reflected in the following: the stenotic lesion can be partially dilated, which reduces the damage to the drug coating of the balloon during balloon transport to the lesion and thus decreases the amount of drug loss; and the lesion plaque is compressed, which increases the degree of balloon apposition and ensures uniform local drug distribution (75). More interestingly, Dr. Robert A. Byrne noted that morphologically intact vessel walls impede drug penetration, while moderate plaque and vessel wall damage can facilitate antiproliferative drug delivery, as well as tissue retention (76). This view also indirectly indicates the importance of preoperative lesion preparation for DCB angioplasty (77).

Although preclinical data have suggested the importance of preoperative lesion preparation for DCB angioplasty (44, 78, 79) and that the preexpansion of scored and cut balloons improves clinical outcomes for DCB angioplasty (75, 80), data from these clinical trials are limited, and clinical data on postoperative complications are inadequate. Compared to conventional balloon angioplasty, scoring and cutting balloons rupture the plaque and cause a higher degree of intimal rupture at a relatively low inflation pressure, improving vascular compliance and allowing for better dilatation of the DCB (81), but coronary atherosclerotic plaques are usually eccentric and heterogeneous (82), and it is worth discussing whether scoring and cutting balloons, although good at creating cracks in the plaque, damage the normal intima and are more likely to cause the incidence of vascular entrapment.

### Double antiplatelet treatment is an important factor affecting the treatment effect of drug-coated balloon

At present, antiplatelet therapy has become the essential drug after PCI, which is currently combined with aspirin and P2Y12 receptor inhibitors for the prevention of stent thrombosis and secondary prevention of ischemic thrombotic events (83, 84). With the improvement of PCI technology, the guidelines on DAPT are also changing (85). Currently, clinical trials on DAPT are mainly comparing two strategies of shortening and prolonging DAPT after PCI. Clinicians face many difficulties in making decisions about the optimal duration of DAPT in order to minimize the risk of ischemic and bleeding complications (86). A relatively systematic overview of clinical guidelines for DAPT duration after drug-eluting stenting is currently presented (87, 88). However, there is no consensus on the duration of DAPT treatment after DCB. Compared with DES, implantation without a foreign body seems to be an obvious advantage of DCB. There is no inflammation caused by the implantation of foreign body (89). Theoretically, the DAPT time after the implantation of DCB should be shorter than that of the DES group. In addition, there lacks clinical trials on the duration of DAPT after DCB application, so we can only collect relevant information from clinical trials of DCB in the treatment of different coronary artery lesions.

Compared with small vessel and large vessel angiogenesis coronary artery disease and bifurcation disease, DCB has good efficacy and good safety in the treatment of ISR (90). In clinical trials of drug-eluting stent restenosis, such as PEPCAD-DES, ISAR-Desire, PEPCAD China ISR, ISAR-Desire, and other clinical trials, the duration of DAPT is generally between 6 and 12 months, and the endpoint of DCB group is better than that of the POBA group (75, 91–93). However, it is interesting that in the clinical trial of Ribs IV, DCB did not perform as well as EES. The DAPT duration in the Ribs IV group was 3 months, while that in the EES group was 12 months (94). Whether the duration of DAPT has an important effect on the clinical trial is worth further investigation. For small vessel and large vessel coronary artery lesions, as well as bifurcation lesions, the current clinical trial data are not very sufficient, In the Basket-Small, BELLO, and other trials of SMALL coronary artery disease, DAPT for stable angina was 1 month in the DCB group and 6 or 12 months in the DES group, but the rates of MACE were similar between these two groups. There were no significant differences in the rates of major bleeding and restenosis between them. Thus, short-term DAPT treatment after DCB for small coronary vessels is safe and effective (95, 96). But according to some limited clinical trials, for patients with short-term DAPT duration, the endpoint of the DCB group was not better than that of the DES group. However, these results showed that short-term DAPT is feasible and safe (97, 98). Compared with long-term DAPT, more clinical trials are needed to verify the optimal duration of DAPT.

## The efficiency of drug-coated balloons in clinical trials

In 2003, a clinical study of DCBs in the treatment of ISR called PACCOCATH was launched. A series of studies and experiments have been performed over the past 20 years. Initially, DCBs were mainly used to treat ISR using bare-metal stents (BMSs) and DESs. With the gradual increase in randomized clinical data, DCBs have been found to be effective in the treatment of emerging small vessel disease, bifurcation disease, large vessel disease, acute coronary syndrome, etc. Therefore, the clinical indications for DCBs are constantly expanding, and DCB technologies are also constantly improving (77, 99, 100). Table 1 shows a comparison of characteristics between DCBs and DES.

**TABLE 1:** Characteristics comparison between DCB and DES.

DCB		DES	
Advantages	Limitations	Advantages	Limitations
No permanent prosthesis	Downstream microembolism	Well targeted	In-stent restenosis
Allows for adaptive remodeling	Elastic recoil	High local tissue concentration	Not suitable for small vascular lesions
Multiple balloon use	Negative vascular remodeling	Systematic side effects are minimal	Fringe effect
Preserves the original anatomy of the vessel	Drug loss during transit		The stent is not attached to the vessel wall

### Factors influencing the efficiency of drug-coated balloons for the treatment of in-stent restenosis

The data from several clinical trials in recent years are shown in Table 2. Compared with BMS, DESs have additional polymeric and drug coatings that could effectively decrease the incidence of ISR (101). Compared with DES, the advantage of DCBs is that they allow for a more uniform distribution of the drug on the surface of the vessel with no metal stent implanted. Therefore, DCBs do not cause delayed healing of the vessel. In consideration of the encouraging results of clinical trials of DCBs for the treatment of ISR, the European Society of Cardiology recommended the use of DCBs for various ISRs in 2014 (4, 102). From a series of larger clinical trials of DCBs for the treatment of ISR in recent years (91, 103–106), it is clear that the MACEs and TLR data of patients treated with DCB are more favorable than those from the plain old balloon angioplasty (POBA) and DES groups in both patients with BMS-ISR and DES-ISR. Interestingly, according to a clinical meta-analysis, DCB angioplasty was comparable to DES repeated stenting in the treatment of BMS-ISR, whereas DCB angioplasty was less effective than DES repeated stenting in the treatment of DES-ISR (107). Previous pathological studies have demonstrated that restenosis after DES was caused by neointimal hyperplasia, as well as neoatherosclerosis, whereas restenosis within BMS stenosis was mainly caused by neointimal hyperplasia (108).

**TABLE 2:** Randomized, controlled trials of the efficiency of DCBs for the treatment of ISR.

Trial name	DCB					DES			Endpoint	
	Name	Drug	Coating Method	Excipient	Dose (μ g/mm^2^)	Name	Drug	Dose (μ g/mm^2^)	MACE (%)	TLR (%)
PEPCAD CHINA ISR (93)	SeQuent Please	PTX	Matrix coating: paclitaxel + hydrophilic spacer	iopromide	3	Taxus Liberté	PTX	1.0	16.5 vs. 16 (TLF)	15.6 vs. 12.3
RIBS IV (94)	SeQuent Please	PTX	Matrix coating: paclitaxel + hydrophilic spacer	iopromide	3	Xience Prime	everolimus	1.0	18 vs. 10	13.0 vs. 4.5
RESTORE (109) ^C^	SeQuent Please	PTX	Matrix coating: paclitaxel + hydrophilic spacer	iopromide	3	Xience Prime	everolimus	1.0	7.0 vs. 4.7	5.8 vs. 1.2
ISAR DESIRE III (92)	SeQuent Please	PTX	Matrix coating: paclitaxel + hydrophilic spacer	iopromide	3	Taxus Liberté	PTX	1.0	23.5 vs. 19.3	22.1 vs. 13.5

PTX, paclitaxel; MACE, major adverse clinical events; TLR, target lesion revascularization.

Drug-coated balloons (DCBs) performed no better than DES regarding TLR and MACEs. In both the RIBS IV and restore clinical trials, which compared everolimus-eluting stents to DCBs coated with paclitaxel, the DES group had better MACEs and TLR than the DCB group in both trials (94, 109). Dr. Fernando Alfonso’s study could explain the results. It was illustrated that patients with DES-ISR might have developed some degree of resistance to antiproliferative drugs (110); with the addition of the neovascular plaques appearing in DES-ISR (108), it might be less effective than a conventional dose of PTX-coated balloon for ISR compared with an eluting stent coated with a new antiproliferative drug. In the PEPCAD CHINA and IASR-DESIRE trials, there was no significant difference in MACEs between the PTX-coated balloon (PCB) and PTX-eluting stent (PES) groups, while the TLR was higher in the DCB group than in the DES group in the IASR-DESIRE trial. It was explained by Dr. Robert A Byrne that the second stenting in the DES group reduced the success of the reintervention (92, 93). In addition, compared to DCBs, DESs undergo less drug loss when delivered to the lesion site. In addition, repeated stenting could achieve a satisfactory drug dose for the specific pathological changes in DES-ISR.

### Factors influencing the efficiency of drug-coated balloon for the treatment of small vessel *de novo* coronary artery disease

Currently, the incidence of stenosis increases with small vessel *de novo* coronary artery disease treated with DES, and the treatment of small vessels remains a great challenge for interventional cardiologists. One of the advantages of DCBs over DES for the treatment of small coronary arteries is the shortened time of dual antiplatelet therapy (DAPT) and the decreased incidence of bleeding-related complications (95, 111, 112), leading to the development of clinical trials of DCBs in this field. However, the results of all current randomized, clinical trials on DCBs for the treatment of small vessel *de novo* coronary artery disease have been inconsistent, mainly because of the heterogeneity in the definition of small vessels, implantation techniques, and measurement results (113). A meta-analysis illustrated that DCBs were not as effective as DES in the treatment of small vessel diseases (114). Moreover, small vessels treated by DCB balloon angioplasty are more prone to restenosis (104). As shown in Table 3, there was no statistically significant difference in TLR or MACEs between the DES and DCB groups, but the restenosis rate was higher in the DCB group than in the DES group. The main causes of restenosis include *de novo* intimal hyperplasia, early vascular recoil, negative vascular remodeling (115–119), and abrupt vessel closure caused by elastic recoil and occlusive plaque. Dr. Yida Tang found that dilation pressure correlated with each endpoint in the DCB group by comparing the dilation pressure of the BELLO trials (112). Higher dilation pressure is more likely to cause acute occlusion of the vessel, while lower dilation pressure not only decreases the tight fit of the balloon to the vessel surface but also results in a lower drug delivery rate. Thus, proper dilation pressure is necessary for the treatment of small vessel diseases. In addition, PTX on DCBs is generally deposited on the arterial wall in the form of crystals, which can maintain the storage of PTX and thus produce an antiproliferation effect (46, 120).

**TABLE 3:** Randomized, controlled trials of the efficiency of DCBs for the treatment of small vessel *de novo* coronary artery disease.

Trial name	DCB				DES			Endpoint		
	Name	Drug	Excipient	Coating Method	Name	Drug	Dose (μ g/mm^2^)	MACE (%)	TLR (%)	Restenosis (%)
PICCOLETO (139)	Dior	PTX	shellolic acid	1:1 mixture of aleuritic and shellolic acid with paclitaxel	TAXUS Liberté	PTX	1.0	35.7 vs. 13.8	32.1 vs. 10.3	32.1 vs. 10.3
BELLO (96)	IN. PACT Falcon	PTX	urea	Crystalline coating: paclitaxel + urea	TAXUS Liberté	PTX	1.0	10.0 vs. 16.3	4.4 vs. 7.6	10.0 vs. 12.4
RESTORE SVD (112)	Restore	PTX	Shellac	Shellac	Resolute Integrity	zotarolimus	1.6	9.6 vs. 9.6	4.4 vs. 2.6	11.0 vs. 7.0
BASKET- SMALL 2 (95)	Sequent Please	PTX	iopromide	Matrix coating: paclitaxel + hydrophilic spacer	TAXUS Element and Xience	zotarolimus PTX and everolimus	1.6 0r 1.0	8 vs. 8	3.4 vs. 4.5	20.4 vs. 21.5
PICCOLETO II (140)	Elutax SV	PTX		Two layers of paclitaxel (the first on the inflated balloon and the second as a crystal power), without any excipient	Xience	everolimus	1.0	N/A	5.6 vs. 5.6	6.3 vs. 6.5

PTX, paclitaxel; MACE, major adverse clinical events; TLR, target lesion revascularization; N/A, not applicable.

What is more interesting is the recent clinical trial conducted in China on the treatment of small vessels with a new drug-coated balloon. The endpoints of the DCB and POBA groups were compared, and the DCB group was significantly better than POBA. In this clinical trial, besides the balloon being an innovation point, the endpoint added an innovative indicator, “LLE (Late luminal enlargement)” (121). There has been a positive correlation between LLE and the therapeutic effect of DCBs in the literature, and LLE may be an important indicator of positive vascular remodeling (122).

### Influencing the efficiency of drug-coated balloons for the treatment of large vessel and bifurcation *de novo* coronary artery disease

Limited clinical trials have been conducted on DCBs in the treatment of large vessel disease (Table 4). Theoretically, large vessels are elastic vessels that are prone to recoil, and they tend to form acute occlusion after DCB application (123). Some clinical trials have evaluated the risk of acute occlusion in large vessels and demonstrated the preferable feasibility of a DCB-only strategy under these circumstances (124, 125). Dr. Raban V. Jege explained that this outcome might be due to the lack of foreign body implantation, as well as its inherent thrombosis (7). There are currently many limitations to DCBs for the treatment of macrovascular diseases, such as pressure control and balanced drug delivery. More clinical trials are required to identify these issues.

**TABLE 4:** Randomized, controlled trials of the efficiency of DCBs for the treatment of large vessel *de novo* coronary artery disease.

Trial name		DCB				DES			Endpoint		
		
	RVD (mm)	Name	Drug	Coating Method	Inflation pressure (atm)	Name	Drug	Inflation Pressure (atm)	Mace (%)	TLR (%)	ST, N
REVELATION (97)	3.28 ± 0.52 vs3.20 ± 0.48	Pantera Lux	Paclitaxel	Paclitaxel + butyryl-trihexyl citrate	10.2 ± 2.7	Xience	everolimus		3 vs. 2	N/A	

DEBUT (98)	2.69 ± 0.45 vs. 2.92 ± 0.31	SeQuent Please	Paclitaxel	Matrix coating: paclitaxel + hydrophilic spacer		Integrity	Bare metal		4 vs. 14**	2 vs. 6	0 vs. 2
David Gobić et al. (141)	2.61 ± 0.49 vs. 3.04 ± 0.46	SeQuent Please	Paclitaxel	Matrix coating: paclitaxel + hydrophilic spacer	8–9	Cobalt-chromium	sirolimus		7.3 vs. 0	N/A	

**P < 0.05 vs. non-DCB group, RVD, reference vessel diameter; TLR, target lesion revascularization; ST, stent thrombosis including definite and possible; N/A, not applicable.

According to previous statistics, coronary bifurcation accounts for approximately 15% to 20% of coronary interventions (126). Unlike other lesions, bifurcation lesions involve both the main and side branches, and the special anatomy of the lesion at a bifurcation increases the difficulties of PCI procedures. When a stent is implanted into the main branch, the plaque in the vessel could be displaced and transferred to the side branch, and the branch vessel could become occluded (127, 128). Currently, the main treatment methods include single stent implantation, double stent implantation, and balloon dilation (7). In the PEPCad-BIF clinical trial, the advantages of DCBs for the treatment of side branches were investigated. Patients were followed up for 9 months, and the DCB group showed significantly better data than the POBA group (129). No consensus has been reached on the treatment of bifurcation lesions due to few clinical trials on DCBs for bifurcation lesions. However, drug dose and dilatation pressure seem to be important factors for DCBs in the treatment of bifurcation lesions.

## Some novel drug-coated balloons designed to improve drug-coated balloon efficiency

Although DCB technologies have been updated over the past 20 years, there still exist some significant limitations, such as vascular perforation, embolism, dissection, and other postoperative complications, greatly attracting people’s attention (130–132). The chocolate touch DCB is a new DCB with a nickel–titanium alloy expansion component. When the balloon expands, the special structure exerts a uniform force upon the vessel, correspondingly decreasing intimal injury. In a recent clinical trial, it was encouraging that the chocolate touch DCB was shown to have great efficacy and safety in the short term (133). Currently, on the market, the drug dose on the surface of PTX-coated balloons is generally 2–3.5 μg/mm^2^. However, in a recent study by Ole Gemeinhardt, the dose of PTX was increased to 6 μg/mm^2^. Experimental data showed that the dose of PTX delivered from this new DCB to the wall of the vessel was two times that of the conventional DCB (134, 135).

In current interventional procedures, it seems difficult for the balloon to fit closely with the vessel wall without injuring the natural anatomy of the vessel. Stephanie Bienek proposed a new type of DCB catheter (HCDCB), which contains a stretchable biocompatible elastic substance. As a compliant balloon, the shrinking surface of this novel DCB is smaller, the drug density on the surface is higher, the drug loss during propulsion is less, and a smaller inflating pressure could achieve a similar inflating effect to that of a commercial DCB at a higher pressure (136, 137).

The improvement of the therapeutic effect of DCBs not only depends on the DCB itself but also lies in the combination of DCBs and other PCI technologies. Junying Kong proposed encouraging results in his study that combined cutting balloons and DCBs (138).

## Conclusion

DCBs implement the concept of “no implant,” avoiding many adverse effects, such as delayed healing caused by exogenous implants. In the early stage, DCBs were mainly applied to treat ISR. Since 2003, increasing clinical trial data have demonstrated the safety and effectiveness of DCBs. Indications for DCBs now extend to ISR for metal stents, small vascular disease, bifurcation disease, large vascular disease, acute coronary syndrome (ACS), and high bleeding risk. The coating of DCBs could exert a therapeutic effect by transferring a proper dose of the drug to vascular lesions in a controlled fashion. However, the instability of the crystal coating could lead to vascular embolism, placing more stringent requirements on the ratio of drugs to excipients and coating spray techniques. In addition, pressure and temperature are two other key factors affecting the efficiency of DCBs. Coronary anatomic factors could also affect the drug delivery efficiency of DCBs. A series of clinical trials of DCBs for different types of lesions have been underway, providing new data and ideas for DCB improvement. The current research on DCBs is not adequate, and more in-depth studies are necessary for future.

## Author contributions

ZC, JL, ZF, and YF structured the manuscript and contributed to the tables and figures. XZ and XJ revised the manuscript and confirmed the final revision. All authors contributed to the manuscript preparation.

## References

[1] Alfonso F Scheller B. State of the art: balloon catheter technologies – Drug-coated balloon. *Eurointervention.* (2017) 13:680–95. 10.4244/EIJ-D-17-00494 28844030

[2] Waksman R Pakala R. Drug-eluting balloon: the comeback kid? *Circ Cardiovasc Interv.* (2009) 2:352–8. 10.1161/CIRCINTERVENTIONS.109.873703 20031739

[3] Cortese B Piraino D Buccheri D Alfonso F. Treatment of bifurcation lesions with drug-coated balloons: a review of currently available scientific data. *Int J Cardiol.* (2016) 220:589–94. 10.1016/j.ijcard.2016.06.079 27390995

[4] Joner M Finn AV Farb A Mont EK Kolodgie FD Ladich E Pathology of drug-eluting stents in humans: delayed healing and late thrombotic risk. *J Am Coll Cardiol.* (2006) 48:193–202. 10.1016/j.jacc.2006.03.042 16814667

[5] Nestelberger T Kaiser C Jeger R. Drug-coated balloons in cardiovascular disease: benefits, challenges, and clinical applications. *Expert Opin Drug Deliv.* (2020) 17:201–11. 10.1080/17425247.2020.1714590 31918593

[6] Schulz A Hauschild T Kleber FX. Treatment of coronary de novo bifurcation lesions with DCB only strategy. *Clin Res Cardiol.* (2014) 103:451–6. 10.1007/s00392-014-0671-9 24522798

[7] Jeger RV Eccleshall S Wan Ahmad WA Ge J Poerner TC Shin E-S Drug-coated balloons for coronary artery disease: third report of the International DCB Consensus Group. *JACC Cardiovasc Interv.* (2020) 13:1391–402. 10.1016/j.jcin.2020.02.043 32473887

[8] Chen Y Gao L Qin Q Chen S Zhang J Chen H Comparison of 2 different drug-coated balloons in in-stent restenosis: the RESTORE ISR China randomized trial. *JACC Cardiovasc Interv.* (2018) 11:2368–77. 10.1016/j.jcin.2018.09.010 30522665

[9] Herdeg C Oberhoff M Baumbach A Blattner A Axel DI Schröder S Local paclitaxel delivery for the prevention of restenosis: biological effects and efficacy in vivo. *J Am Coll Cardiol.* (2000) 35:1969–76. 10.1016/s0735-1097(00)00614-8 10841250

[10] Kolachalama VB Pacetti SD Franses JW Stankus JJ Zhao HQ Shazly T Mechanisms of tissue uptake and retention in zotarolimus-coated balloon therapy. *Circulation.* (2013) 127:2047–55. 10.1161/CIRCULATIONAHA.113.002051 23584359 PMC3748613

[11] Schorn I Malinoff H Anderson S Lecy C Wang J Giorgianni J The lutonix^®^ drug-coated balloon: a novel drug delivery technology for the treatment of vascular disease. *Adv Drug Deliv Rev.* (2017) 112:78–87. 10.1016/j.addr.2017.05.015 28559093

[12] Gray WA Granada JF. Drug-coated balloons for the prevention of vascular restenosis. *Circulation.* (2010) 121:2672–80. 20566965 10.1161/CIRCULATIONAHA.110.936922

[13] Zhu L Chen L. Progress in research on paclitaxel and tumor immunotherapy. *Cell Mol Biol Lett.* (2019) 24:40. 10.1186/s11658-019-0164-y 31223315 PMC6567594

[14] Chowdhury MM Singh K Albaghdadi MS Khraishah H Mauskapf A Kessinger CW Paclitaxel drug-coated balloon angioplasty suppresses progression and inflammation of experimental atherosclerosis in rabbits. *JACC Basic Transl Sci.* (2020) 5:685–95. 10.1016/j.jacbts.2020.04.007 32760856 PMC7393431

[15] Granada JF Stenoien M Buszman PP Tellez A Langanki D Kaluza GL Mechanisms of tissue uptake and retention of paclitaxel-coated balloons: impact on neointimal proliferation and healing. *Open Heart.* (2014) 1:e000117. 10.1136/openhrt-2014-000117 25332821 PMC4189287

[16] Axel DI Kunert W Göggelmann C Oberhoff M Herdeg C Küttner A Paclitaxel inhibits arterial smooth muscle cell proliferation and migration in vitro and in vivo using local drug delivery. *Circulation.* (1997) 96:636–45. 10.1161/01.CIR.96.2.636 9244237

[17] Kamath KR Barry JJ Miller KM. The Taxus drug-eluting stent: a new paradigm in controlled drug delivery. *Adv Drug Deliv Rev.* (2006) 58:412–36. 10.1016/j.addr.2006.01.023 16647782

[18] Sollott SJ Cheng L Pauly RR Jenkins GM Monticone RE Kuzuya M Taxol inhibits neointimal smooth muscle cell accumulation after angioplasty in the rat. *J Clin Invest.* (1995) 95:1869–76. 10.1172/JCI117867 7706494 PMC295730

[19] Schneider PA Laird JR Doros G Gao Q Ansel G Brodmann M Mortality not correlated with paclitaxel exposure: an independent patient-level meta-analysis of a drug-coated balloon. *J Am Coll Cardiol.* (2019) 73:2550–63. 10.1016/j.jvs.2019.06.094 30690141

[20] Hwang C-W Edelman ER. Arterial ultrastructure influences transport of locally delivered drugs. *Circ Res.* (2002) 90:826–32. 10.1161/01.res.0000016672.26000.9e 11964377

[21] Radeleff B Lopez-Benitez R Stampfl U Stampfl S Sommer C Thierjung H Paclitaxel-induced arterial wall toxicity and inflammation: tissue uptake in various dose densities in a minipig model. *J Vasc Interv Radiol.* (2010) 21:1262–70. 10.1016/j.jvir.2010.02.020 20656224

[22] Katsanos K Spiliopoulos S Kitrou P Krokidis M Karnabatidis D. Risk of death following application of paclitaxel-coated balloons and stents in the femoropopliteal artery of the leg: a systematic review and meta-analysis of randomized controlled trials. *J Am Heart Assoc.* (2018) 7:e011245. 10.1161/JAHA.118.011245 PMC6405619 30561254

[23] Zeller T Baumgartner I Scheinert D Brodmann M Bosiers M Micari A Drug-eluting balloon versus standard balloon angioplasty for infrapopliteal arterial revascularization in critical limb ischemia: 12-month results from the IN.PACT DEEP randomized trial. *J Am Coll Cardiol.* (2014) 64:1568–76. 10.1016/j.jacc.2014.06.1198 25301459

[24] Nordanstig J James S Andersson M Andersson M Danielsson P Gillgren P Mortality with paclitaxel-coated devices in peripheral artery disease. *N Engl J Med.* (2020) 383:2538–46. 33296560 10.1056/NEJMoa2005206

[25] Martinet W De Loof H De Meyer GRY. mTOR inhibition: a promising strategy for stabilization of atherosclerotic plaques. *Atherosclerosis.* (2014) 233:601–7. 10.1016/j.atherosclerosis.2014.01.040 24534455

[26] Boada C Zinger A Tsao C Zhao P Martinez JO Hartman K Rapamycin-loaded biomimetic nanoparticles reverse vascular inflammation. *Circ Res.* (2020) 126:25–37. 10.1161/CIRCRESAHA.119.315185 31647755

[27] Fukuda D Sata M Tanaka K Nagai R. Potent inhibitory effect of sirolimus on circulating vascular progenitor cells. *Circulation.* (2005) 111:926–31. 10.1161/01.CIR.0000155612.47040.17 15710768

[28] Hill JM Zalos G Halcox JPJ Schenke WH Waclawiw MA Quyyumi AA Circulating endothelial progenitor cells, vascular function, and cardiovascular risk. *N Engl J Med.* (2003) 348:593–600. 10.1056/NEJMoa022287 12584367

[29] Vasa M Fichtlscherer S Aicher A Adler K Urbich C Martin H Number and migratory activity of circulating endothelial progenitor cells inversely correlate with risk factors for coronary artery disease. *Circ Res.* (2001) 89:E1–7. 10.1161/hh1301.093953 11440984

[30] Clever YP Peters D Calisse J Bettink S Berg M-C Sperling C Novel sirolimus-coated balloon catheter: in vivo evaluation in a porcine coronary model. *Circ Cardiovasc Interv.* (2016) 9:e003543. 10.1161/CIRCINTERVENTIONS.115.003543 27069105

[31] Ali RM Abdul Kader MASK Wan Ahmad WA Ong TK Liew HB Omar A-F Treatment of coronary drug-eluting stent restenosis by a sirolimus- or paclitaxel-coated balloon. *JACC Cardiovasc Interv.* (2019) 12:558–66. 10.1016/j.jcin.2018.11.040 30898253

[32] Ahmad WAW Nuruddin AA Abdul Kader MASK Ong TK Liew HB Ali RM Treatment of coronary De Novo lesions by a sirolimus- or paclitaxel-coated balloon. *JACC Cardiovasc Interv.* (2022) 15:770–9. 10.1016/j.jcin.2022.01.012 35305906

[33] Yang W Ge J Liu H Zhao K Liu X Qu X Arsenic trioxide eluting stent reduces neointima formation in a rabbit iliac artery injury model. *Cardiovasc Res.* (2006) 72:483–93. 10.1016/j.cardiores.2006.08.010 17020754

[34] Chen Y-W Smith ML Sheets M Ballaron S Trevillyan JM Burke SE Zotarolimus, a novel sirolimus analogue with potent anti-proliferative activity on coronary smooth muscle cells and reduced potential for systemic immunosuppression. *J Cardiovasc Pharmacol.* (2007) 49:228–35. 10.1097/FJC.0b013e3180325b0a 17438408

[35] Scheller B Speck U Abramjuk C Bernhardt U Böhm M Nickenig G. Paclitaxel balloon coating, a novel method for prevention and therapy of restenosis. *Circulation.* (2004) 110:810–4. 10.1161/01.CIR.0000138929.71660.E0 15302790

[36] Cremers B Toner JL Schwartz LB von Oepen R Speck U Kaufels N Inhibition of neointimal hyperplasia with a novel zotarolimus coated balloon catheter. *Clin Res Cardiol.* (2012) 101:469–76. 10.1007/s00392-012-0415-7 22293991

[37] Valgimigli M Patialiakas A Thury A McFadden E Colangelo S Campo G Zotarolimus-eluting versus bare-metal stents in uncertain drug-eluting stent candidates. *J Am Coll Cardiol.* (2015) 65:805–15. 10.1016/j.jacc.2014.11.053 25720624

[38] Buiten RA Ploumen EH Zocca P Doggen CJM Jessurun GAJ Schotborgh CE Thin composite-wire-strut zotarolimus-eluting stents versus ultrathin-strut sirolimus-eluting stents in BIONYX at 2 years. *JACC Cardiovasc Interv.* (2020) 13:1100–9. 10.1016/j.jcin.2020.01.230 32381186

[39] Xu B Yang Y Yuan Z Du Z Wong SC Généreux P Zotarolimus- and paclitaxel-eluting stents in an all-comer population in China: the RESOLUTE China randomized controlled trial. *JACC Cardiovasc Interv.* (2013) 6:664–70. 10.1016/j.jcin.2013.03.001 23523240

[40] Tzafriri AR Vukmirovic N Kolachalama VB Astafieva I Edelman ER. Lesion complexity determines arterial drug distribution after local drug delivery. *J Control Release.* (2010) 142:332–8. 10.1016/j.jconrel.2009.11.007 19925836 PMC2994187

[41] Scheller B Speck U Romeike B Schmitt A Sovak M Böhm M Contrast media as carriers for local drug delivery. Successful inhibition of neointimal proliferation in the porcine coronary stent model. *Eur Heart J.* (2003) 24:1462–7. 10.1016/s0195-668x(03)00317-8 12909076

[42] Scheller B Speck U Schmitt A Böhm M Nickenig G. Addition of paclitaxel to contrast media prevents restenosis after coronary stent implantation. *J Am Coll Cardiol.* (2003) 42:1415–20. 14563585 10.1016/s0735-1097(03)01056-8

[43] Colleran R Joner M Kufner S Altevogt F Neumann F-J Abdel-Wahab M Comparative efficacy of two paclitaxel-coated balloons with different excipient coatings in patients with coronary in-stent restenosis: a pooled analysis of the intracoronary stenting and angiographic results: optimizing treatment of drug eluting stent in-stent restenosis 3 and 4 (ISAR-DESIRE 3 and ISAR-DESIRE 4) trials. *Int J Cardiol.* (2018) 252:57–62. 10.1016/j.ijcard.2017.11.076 29203209

[44] Radke PW Joner M Joost A Byrne RA Hartwig S Bayer G Vascular effects of paclitaxel following drug-eluting balloon angioplasty in a porcine coronary model: the importance of excipients. *Eurointervention.* (2011) 7:730–7. 10.4244/EIJV7I6A116 21986331

[45] Anderson JA Remund T Pohlson K Lamichhane S Evans C Evans R In vitro and in vivo evaluation of effect of excipients in local delivery of paclitaxel using microporous infusion balloon catheters. *J Biomed Mater Res B Appl Biomater.* (2017) 105:376–90. 10.1002/jbm.b.33564 26513737

[46] Kolodgie FD Pacheco E Yahagi K Mori H Ladich E Virmani R. Comparison of particulate embolization after femoral artery treatment with IN.PACT admiral versus lutonix 035 Paclitaxel-coated balloons in healthy swine. *J Vasc Interv Radiol.* (2016) 27:1676–85.e2. 10.1016/j.jvir.2016.06.036 27641674

[47] Tesfamariam B. Local arterial wall drug delivery using balloon catheter system. *J Control Release.* (2016) 238:149–56. 27473765 10.1016/j.jconrel.2016.07.041

[48] Kaule S Minrath I Stein F Kragl U Schmidt W Schmitz K-P Correlating coating characteristics with the performance of drug-coated balloons–a comparative in vitro investigation of own established hydrogel- and ionic liquid-based coating matrices. *PLoS One.* (2015) 10:e0116080. 10.1371/journal.pone.0116080 25734818 PMC4348426

[49] Pan CJ Tang JJ Weng YJ Wang J Huang N. Preparation, characterization and anticoagulation of curcumin-eluting controlled biodegradable coating stents. *J Control Release.* (2006) 116:42–9. 10.1016/j.jconrel.2006.08.023 17046093

[50] Tzafriri AR Muraj B Garcia-Polite F Salazar-Martín AG Markham P Zani B Balloon-based drug coating delivery to the artery wall is dictated by coating micro-morphology and angioplasty pressure gradients. *Biomaterials.* (2020) 260:120337. 10.1016/j.biomaterials.2020.120337 32937269 PMC7530113

[51] Stolzenburg N Breinl J Bienek S Jaguszewski M Löchel M Taupitz M Paclitaxel-coated balloons: investigation of drug transfer in healthy and atherosclerotic arteries – First experimental results in rabbits at low inflation pressure. *Cardiovasc Drugs Ther.* (2016) 30:263–70. 10.1007/s10557-016-6658-1 27033233 PMC4919377

[52] Delrio FW de Boer MP Knapp JA David Reedy E Clews PJ Dunn ML. The role of van der Waals forces in adhesion of micromachined surfaces. *Nat Mater.* (2005) 4:629–34. 10.1038/nmat1431 16025121

[53] Holmes DR Vlietstra RE Smith HC Vetrovec GW Kent KM Cowley MJ Restenosis after percutaneous transluminal coronary angioplasty (PTCA): a report from the PTCA Registry of the National Heart, Lung, and Blood Institute. *Am J Cardiol.* (1984) 53:77C–81C. 10.1016/0002-9149(84)90752-5 6233894

[54] Fakhraei Lahiji S Kim Y Kang G Kim S Lee S Jung H. Tissue interlocking dissolving microneedles for accurate and efficient transdermal delivery of biomolecules. *Sci Rep.* (2019) 9:7886. 10.1038/s41598-019-44418-6 31133711 PMC6536679

[55] Yadav PR Pattanayek SK. Modulation of physicochemical properties of polymers for effective insulin delivery systems. In: Chandra P Pandey L editors. *Biointerface Engineering: Prospects in Medical Diagnostics and Drug Delivery.* Singapore: Springer (2020). 10.1007/978-981-15-4790-4_6

[56] Wang M Hu L Xu C. Recent advances in the design of polymeric microneedles for transdermal drug delivery and biosensing. *Lab Chip.* (2017) 17:1373–87. 10.1039/c7lc00016b 28352876

[57] Ye Y Yu J Wen D Kahkoska AR Gu Z. Polymeric microneedles for transdermal protein delivery. *Adv Drug Deliv Rev.* (2018) 127:106–18. 10.1016/j.addr.2018.01.015 29408182 PMC6020694

[58] Lee K Lee J Lee SG Park S Yang DS Lee J-J Microneedle drug eluting balloon for enhanced drug delivery to vascular tissue. *J Control Release.* (2020) 321:174–83. 10.1016/j.jconrel.2020.02.012 32035908

[59] Van Renterghem J Dhondt H Verstraete G De Bruyne M Vervaet C De Beer T. The impact of the injection mold temperature upon polymer crystallization and resulting drug release from immediate and sustained release tablets. *Int J Pharm.* (2018) 541:108–16. 10.1016/j.ijpharm.2018.01.053 29409747

[60] Weihe WH. The effect of temperature on the action of drugs. *Annu Rev Pharmacol.* (1973) 13:409–25. 10.1146/annurev.pa.13.040173.002205 4576895

[61] Arenas J Perez JJ Trujillo M Berjano E. Computer modeling and ex vivo experiments with a (saline-linked) irrigated electrode for RF-assisted heating. *Biomed Eng Online.* (2014) 13:164. 10.1186/1475-925X-13-164 25494912 PMC4271499

[62] González-Suárez A Trujillo M Burdío F Andaluz A Berjano E. Could the heat sink effect of blood flow inside large vessels protect the vessel wall from thermal damage during RF-assisted surgical resection? *Med Phys.* (2014) 41:083301. 10.1118/1.4890103 25086561

[63] Zorbas G Samaras T. A study of the sink effect by blood vessels in radiofrequency ablation. *Comput Biol Med.* (2015) 57:182–6. 25575184 10.1016/j.compbiomed.2014.12.014

[64] Fram DB Aretz TA Mikan JF Raisner A Mitchel JF Gillam LD In vivo radiofrequency thermal balloon angioplasty of porcine coronary arteries: histologic effects and safety. *Am Heart J.* (1993) 126:969–78. 10.1016/0002-8703(93)90714-k 8213457

[65] Buller CE Culp SC Sketch MH Phillips HR Virmani R Stack RS. Thermal-perfusion balloon coronary angioplasty: in vivo evaluation. *Am Heart J.* (1993) 125:226–33. 10.1016/0002-8703(93)90079-o 8417522

[66] Gurbel PA Anderson RD. New concept in coronary angioplasty: dilatation with a helical balloon that allows simultaneous autoperfusion. *Cathet Cardiovasc Diagn.* (1997) 40:109–16. 10.1002/(sici)1097-0304(199701)40:1<109::aid-ccd21>3.0.co;2-m 8993827

[67] De Maria GL Scarsini R Banning AP. Management of calcific coronary artery lesions: is it time to change our interventional therapeutic approach? *JACC Cardiovasc Interv.* (2019) 12:1465–78. 10.1016/j.jcin.2019.03.038 31395217

[68] Anbalakan K Toh HW Ang HY Buist ML Leo HL. Assessing the influence of atherosclerosis on drug coated balloon therapy using computational modelling. *Eur J Pharm Biopharm.* (2021) 158:72–82. 10.1016/j.ejpb.2020.09.016 33075477

[69] Fernández-Parra R Laborda A Lahuerta C Lostalé F Aramayona J de Blas I Pharmacokinetic study of paclitaxel concentration after drug-eluting balloon angioplasty in the iliac artery of healthy and atherosclerotic rabbit models. *J Vasc Interv Radiol.* (2015) 26:1380–7.e1. 10.1016/j.jvir.2015.05.022 26190185

[70] Finn AV Nakazawa G Joner M Kolodgie FD Mont EK Gold HK Vascular responses to drug eluting stents: importance of delayed healing. *Arterioscler Thromb Vasc Biol.* (2007) 27:1500–10. 10.1161/ATVBAHA.107.144220 17510464

[71] Lagerqvist B James SK Stenestrand U Lindbäck J Nilsson T Wallentin L. Long-term outcomes with drug-eluting stents versus bare-metal stents in Sweden. *N Engl J Med.* (2007) 356:1009–19. 10.1056/NEJMoa067722 17296822

[72] Kastrati A Dibra A Mehilli J Mayer S Pinieck S Pache J Predictive factors of restenosis after coronary implantation of sirolimus- or paclitaxel-eluting stents. *Circulation.* (2006) 113:2293–300. 10.1161/CIRCULATIONAHA.105.601823 16682614

[73] Fanelli F Cannavale A Gazzetti M Lucatelli P Wlderk A Cirelli C Calcium burden assessment and impact on drug-eluting balloons in peripheral arterial disease. *Cardiovasc Intervent Radiol.* (2014) 37:898–907. 10.1007/s00270-014-0904-3 24806955

[74] Sirianni RW Kremer J Guler I Chen Y-L Keeley FW Saltzman WM. Effect of extracellular matrix elements on the transport of paclitaxel through an arterial wall tissue mimic. *Biomacromolecules.* (2008) 9:2792–8. 10.1021/bm800571s 18785771

[75] Kufner S Joner M Schneider S Tölg R Zrenner B Repp J Neointimal modification with scoring balloon and efficacy of drug-coated balloon therapy in patients with restenosis in drug-eluting coronary stents: a randomized controlled trial. *JACC Cardiovasc Interv.* (2017) 10:1332–40. 10.1016/j.jcin.2017.04.024 28683939

[76] Byrne RA Joner M Alfonso F Kastrati A. Drug-coated balloon therapy in coronary and peripheral artery disease. *Nat Rev Cardiol.* (2014) 11:13–23. 10.1038/nrcardio.2013.165 24189405

[77] Kleber FX Rittger H Bonaventura K Zeymer U Wöhrle J Jeger R Drug-coated balloons for treatment of coronary artery disease: updated recommendations from a consensus group. *Clin Res Cardiol.* (2013) 102:785–97. 10.1007/s00392-013-0609-7 23982467

[78] Joner M Byrne RA Lapointe JM Radke PW Bayer G Steigerwald K Comparative assessment of drug-eluting balloons in an advanced porcine model of coronary restenosis. *Thromb Haemost.* (2011) 105:864–72. 10.1160/TH10-11-0698 21301785

[79] Joner M Radke PW Byrne RA Hartwig S Steigerwald K Leclerc G Preclinical evaluation of a novel drug-eluting balloon in an animal model of in-stent stenosis. *J Biomater Appl.* (2013) 27:717–26. 10.1177/0885328211423784 22262578

[80] Bonaventura K Schwefer M Yusof AKM Waliszewski M Krackhardt F Steen P Systematic scoring balloon lesion preparation for drug-coated balloon angioplasty in clinical routine: results of the PASSWORD observational study. *Adv Ther.* (2020) 37:2210–23. 10.1007/s12325-020-01320-2 32274746 PMC7467461

[81] Jujo K Saito K Ishida I Kim A Suzuki Y Furuki Y Intimal disruption affects drug-eluting cobalt-chromium stent expansion: a randomized trial comparing scoring and conventional balloon predilation. *Int J Cardiol.* (2016) 221:23–31. 10.1016/j.ijcard.2016.07.002 27400292

[82] Yamagishi M Terashima M Awano K Kijima M Nakatani S Daikoku S Morphology of vulnerable coronary plaque: insights from follow-up of patients examined by intravascular ultrasound before an acute coronary syndrome. *J Am Coll Cardiol.* (2000) 35:106–11. 10.1016/s0735-1097(99)00533-1 10636267

[83] Bertrand ME Legrand V Boland J Fleck E Bonnier J Emmanuelson H Randomized multicenter comparison of conventional anticoagulation versus antiplatelet therapy in unplanned and elective coronary stenting. The full anticoagulation versus aspirin and ticlopidine (fantastic) study. *Circulation.* (1998) 98:1597–603. 10.1161/01.cir.98.16.1597 9778323

[84] Steinhubl SR Berger PB Mann JT Fry ETA DeLago A Wilmer C Early and sustained dual oral antiplatelet therapy following percutaneous coronary intervention: a randomized controlled trial. *JAMA.* (2002) 288:2411–20. 10.1001/jama.288.19.2411 12435254

[85] Miyazaki Y Suwannasom P Sotomi Y Abdelghani M Tummala K Katagiri Y Single or dual antiplatelet therapy after PCI. *Nat Rev Cardiol.* (2017) 14:294–303. 10.1038/nrcardio.2017.12 28181585

[86] Moon JY Franchi F Rollini F Angiolillo DJ. The quest for safer antithrombotic treatment regimens in patients with coronary artery disease: new strategies and paradigm shifts. *Expert Rev Hematol.* (2018) 11:5–12. 10.1080/17474086.2018.1400378 29091481

[87] Levine GN Bates ER Bittl JA Brindis RG Fihn SD Fleisher LA 2016 ACC/AHA guideline focused update on duration of dual antiplatelet therapy in patients with coronary artery disease: a report of the American College of Cardiology/American Heart Association task force on clinical practice guidelines. *J Am Coll Cardiol.* (2016) 68:1082–115. 27036918 10.1016/j.jacc.2016.03.513

[88] Valgimigli M Bueno H Byrne RA Collet J-P Costa F Jeppsson A 2017 ESC focused update on dual antiplatelet therapy in coronary artery disease developed in collaboration with EACTS: the task force for dual antiplatelet therapy in coronary artery disease of the European Society of Cardiology (ESC) and of the European Association for Cardio-Thoracic Surgery (EACTS). *Eur Heart J.* (2018) 39:213–60. 10.1093/eurheartj/ehx419 28886622

[89] Zhang Y Zhang X Dong Q Chen D Xu Y Jiang J. Duration of dual antiplatelet therapy after implantation of drug-coated balloon. *Front Cardiovasc Med.* (2021) 8:762391. 10.3389/fcvm.2021.762391 34926613 PMC8671702

[90] Neumann F-J Sousa-Uva M Ahlsson A Alfonso F Banning AP Benedetto U 2018 ESC/EACTS guidelines on myocardial revascularization. *Eur Heart J.* (2019) 40:87–165. 10.1093/eurheartj/ehy855 30165437

[91] Rittger H Brachmann J Sinha A-M Waliszewski M Ohlow M Brugger A A randomized, multicenter, single-blinded trial comparing paclitaxel-coated balloon angioplasty with plain balloon angioplasty in drug-eluting stent restenosis: the PEPCAD-DES study. *J Am Coll Cardiol.* (2012) 59:1377–82. 10.1016/j.jacc.2012.01.015 22386286

[92] Byrne RA Neumann F-J Mehilli J Pinieck S Wolff B Tiroch K Paclitaxel-eluting balloons, paclitaxel-eluting stents, and balloon angioplasty in patients with restenosis after implantation of a drug-eluting stent (ISAR-DESIRE 3): a randomised, open-label trial. *Lancet.* (2013) 381:461–7. 10.1016/S0140-6736(12)61964-3 23206837

[93] Xu B Gao R Wang J Yang Y Chen S Liu B A prospective, multicenter, randomized trial of paclitaxel-coated balloon versus paclitaxel-eluting stent for the treatment of drug-eluting stent in-stent restenosis: results from the PEPCAD China ISR trial. *JACC Cardiovasc Interv.* (2014) 7:204–11. 10.1016/j.jcin.2013.08.011 24556098

[94] Alfonso F Pérez-Vizcayno MJ Cárdenas A García del Blanco B García-Touchard A López-Minguéz JR A prospective randomized trial of drug-eluting balloons versus everolimus-eluting stents in patients with in-stent restenosis of drug-eluting stents: the RIBS IV randomized clinical trial. *J Am Coll Cardiol.* (2015) 66:23–33. 10.1016/j.jacc.2015.04.063 26139054

[95] Jeger RV Farah A Ohlow M-A Mangner N Möbius-Winkler S Leibundgut G Drug-coated balloons for small coronary artery disease (BASKET-SMALL 2): an open-label randomised non-inferiority trial. *Lancet.* (2018) 392:849–56. 10.2139/ssrn.3210892 30170854

[96] Latib A Colombo A Castriota F Micari A Cremonesi A De Felice F A randomized multicenter study comparing a paclitaxel drug-eluting balloon with a paclitaxel-eluting stent in small coronary vessels: the BELLO (Balloon Elution and Late Loss Optimization) study. *J Am Coll Cardiol.* (2012) 60:2473–80. 10.1016/j.jacc.2012.09.020 23158530

[97] Vos NS Fagel ND Amoroso G Herrman J-PR Patterson MS Piers LH Paclitaxel-coated balloon angioplasty versus drug-eluting stent in acute myocardial infarction: the REVELATION randomized trial. *JACC Cardiovasc Interv.* (2019) 12:1691–9. 10.1016/j.jcin.2019.04.016 31126887

[98] Rissanen TT Uskela S Eränen J Mäntylä P Olli A Romppanen H Drug-coated balloon for treatment of de-novo coronary artery lesions in patients with high bleeding risk (DEBUT): a single-blind, randomised, non-inferiority trial. *Lancet.* (2019) 394:230–9. 10.1016/S0140-6736(19)31126-2 31204115

[99] Kleber FX Mathey DG Rittger H Scheller B. How to use the drug-eluting balloon: recommendations by the German consensus group. *Eurointervention.* (2011) 7 (Suppl. K):K125–8. 10.4244/EIJV7SKA21 22027722

[100] Scheller B Hehrlein C Bocksch W Rutsch W Haghi D Dietz U Two year follow-up after treatment of coronary in-stent restenosis with a paclitaxel-coated balloon catheter. *Clin Res Cardiol.* (2008) 97:773–81. 10.1007/s00392-008-0682-5 18536865

[101] Kolandaivelu K Swaminathan R Gibson WJ Kolachalama VB Nguyen-Ehrenreich K-L Giddings VL Stent thrombogenicity early in high-risk interventional settings is driven by stent design and deployment and protected by polymer-drug coatings. *Circulation.* (2011) 123:1400–9. 10.1161/CIRCULATIONAHA.110.003210 21422389 PMC3131199

[102] Windecker S Kolh P Alfonso F Collet J-P Cremer J Falk V 2014 ESC/EACTS guidelines on myocardial revascularization: the task force on myocardial revascularization of the European Society of Cardiology (ESC) and the European Association for Cardio-Thoracic Surgery (EACTS)developed with the special contribution of the European Association of Percutaneous Cardiovascular Interventions (EAPCI). *Eur Heart J.* (2014) 35:2541–619. 25173339 10.1093/eurheartj/ehu278

[103] Habara S Mitsudo K Kadota K Goto T Fujii S Yamamoto H Effectiveness of paclitaxel-eluting balloon catheter in patients with sirolimus-eluting stent restenosis. *JACC Cardiovasc Interv.* (2011) 4:149–54. 21349452 10.1016/j.jcin.2010.10.012

[104] Pleva L Kukla P Kusnierova P Zapletalova J Hlinomaz O. Comparison of the efficacy of paclitaxel-eluting balloon catheters and everolimus-eluting stents in the treatment of coronary in-stent restenosis: the treatment of in-stent restenosis study. *Circ Cardiovasc Interv.* (2016) 9:e003316. 10.1161/CIRCINTERVENTIONS.115.003316 27069104

[105] Scheller B Hehrlein C Bocksch W Rutsch W Haghi D Dietz U Treatment of coronary in-stent restenosis with a paclitaxel-coated balloon catheter. *N Engl J Med.* (2006) 355:2113–24. 10.1056/NEJMoa061254 17101615

[106] Unverdorben M Vallbracht C Cremers B Heuer H Hengstenberg C Maikowski C Paclitaxel-coated balloon catheter versus paclitaxel-coated stent for the treatment of coronary in-stent restenosis. *Circulation.* (2009) 119:2986–94. 10.1161/CIRCULATIONAHA.108.839282 19487593

[107] Giacoppo D Alfonso F Xu B Claessen BEPM Adriaenssens T Jensen C Drug-coated balloon angioplasty versus drug-eluting stent implantation in patients with coronary stent restenosis. *J Am Coll Cardiol.* (2020) 75:2664–78. 10.1016/j.jacc.2020.04.006 32466881

[108] Byrne RA Joner M Kastrati A. Stent thrombosis and restenosis: what have we learned and where are we going? The Andreas Grüntzig Lecture ESC 2014. *Eur Heart J.* (2015) 36:3320–31. 10.1093/eurheartj/ehv511 26417060 PMC4677274

[109] Wong YTA Kang D-Y Lee JB Rha S-W Hong YJ Shin E-S Comparison of drug-eluting stents and drug-coated balloon for the treatment of drug-eluting coronary stent restenosis: a randomized RESTORE trial. *Am Heart J.* (2018) 197:35–42. 10.1016/j.ahj.2017.11.008 29447782

[110] Alfonso F Pérez-Vizcayno MJ García Del Blanco B García-Touchard A López-Mínguez J-R Masotti M Everolimus-eluting stents in patients with bare-metal and drug-eluting in-stent restenosis: results from a patient-level pooled analysis of the RIBS IV and V trials. *Circ Cardiovasc Interv.* (2016) 9:e003479. 10.1161/CIRCINTERVENTIONS.115.003479 27412868

[111] Jeger RV Farah A Ohlow M-A Mangner N Möbius-Winkler S Weilenmann D Long-term efficacy and safety of drug-coated balloons versus drug-eluting stents for small coronary artery disease (BASKET-SMALL 2): 3-year follow-up of a randomised, non-inferiority trial. *Lancet.* (2020) 396:1504–10. 10.1016/S0140-6736(20)32173-5 33091360

[112] Tang Y Qiao S Su X Chen Y Jin Z Chen H Drug-coated balloon versus drug-eluting stent for small-vessel disease: the RESTORE SVD China randomized trial. *JACC Cardiovasc Interv.* (2018) 11:2381–92. 30522667 10.1016/j.jcin.2018.09.009

[113] Giannini F Khokhar AA Albani S. Percutaneous intervention in small-vessel coronary disease: time to clear the fog? *JACC Cardiovasc Interv.* (2020) 13:805–7. 10.1016/j.jcin.2019.11.011 32061603

[114] Siontis GCM Piccolo R Praz F Valgimigli M Räber L Mavridis D Percutaneous coronary interventions for the treatment of stenoses in small coronary arteries: a network meta-analysis. *JACC Cardiovasc Interv.* (2016) 9:1324–34. 10.1016/j.jcin.2016.03.025 27318845

[115] Gertz SD Gimple LW Banai S Ragosta M Powers ER Roberts WC Geometric remodeling is not the principal pathogenetic process in restenosis after balloon angioplasty. Evidence from correlative angiographic-histomorphometric studies of atherosclerotic arteries in rabbits. *Circulation.* (1994) 90:3001–8. 10.1161/01.CIR.90.6.3001 7994848

[116] Strauss BH Chisholm RJ Keeley FW Gotlieb AI Logan RA Armstrong PW. Extracellular matrix remodeling after balloon angioplasty injury in a rabbit model of restenosis. *Circ Res.* (1994) 75:650–8. 10.1161/01.res.75.4.650 7923611

[117] Post MJ Borst C Kuntz RE. The relative importance of arterial remodeling compared with intimal hyperplasia in lumen renarrowing after balloon angioplasty. A study in the normal rabbit and the hypercholesterolemic Yucatan micropig. *Circulation.* (1994) 89:2816–21. 10.1161/01.cir.89.6.2816 8205696

[118] Lincoff AM Popma JJ Ellis SG Hacker JA Topol EJ. Abrupt vessel closure complicating coronary angioplasty: clinical, angiographic and therapeutic profile. *J Am Coll Cardiol.* (1992) 19:926–35. 10.1016/0735-1097(92)90272-O 1552113

[119] Tenaglia AN Fortin DF Califf RM Frid DJ Nelson CL Gardner L Predicting the risk of abrupt vessel closure after angioplasty in an individual patient. *J Am Coll Cardiol.* (1994) 24:1004–11. 10.1016/0735-1097(94)90862-1 7930190

[120] Torii S Yahagi K Mori H Harari E Romero ME Kolodgie FD Safety of zilver PTX drug-eluting stent implantation following drug-coated balloon dilation in a healthy swine model. *J Endovasc Ther.* (2018) 25:118–26. 10.1177/1526602817743747 29161933

[121] Xu K Fu G Tong Q Liu B Han X Zhang J Biolimus-coated balloon in small-vessel coronary artery disease: the BIO-RISE CHINA study. *JACC Cardiovasc Interv.* (2022) 15:1219–26. 10.1016/j.jcin.2022.03.024 35738744

[122] Yamamoto T Sawada T Uzu K Takaya T Kawai H Yasaka Y. Possible mechanism of late lumen enlargement after treatment for de novo coronary lesions with drug-coated balloon. *Int J Cardiol.* (2020) 321:30–7. 10.1016/j.ijcard.2020.07.028 32710988

[123] Wagenseil JE Mecham RP. Vascular extracellular matrix and arterial mechanics. *Physiol Rev.* (2009) 89:957–89. 19584318 10.1152/physrev.00041.2008 PMC2775470

[124] Rosenberg M Waliszewski M Krackhardt F Chin K Wan Ahmad WA Caramanno G Drug coated balloon-only strategy in de novo lesions of large coronary vessels. *J Interv Cardiol.* (2019) 2019:6548696. 10.1155/2019/6548696 31772539 PMC6739788

[125] Yu X Ji F Xu F Zhang W Wang X Lu D Treatment of large de novo coronary lesions with paclitaxel-coated balloon only: results from a Chinese institute. *Clin Res Cardiol.* (2019) 108:234–43. 10.1007/s00392-018-1346-8 30074078

[126] Sawaya FJ Lefèvre T Chevalier B Garot P Hovasse T Morice M-C Contemporary approach to coronary bifurcation lesion treatment. *JACC Cardiovasc Interv.* (2016) 9:1861–78. 10.1016/j.jcin.2016.06.056 27659563

[127] Latib A Colombo A. Bifurcation disease: what do we know, what should we do? *JACC Cardiovasc Interv.* (2008) 1:218–26. 19463303 10.1016/j.jcin.2007.12.008

[128] Colombo A Giannini F. Challenging the “provisional” technique for coronary bifurcation lesions. *JACC Cardiovasc Interv.* (2016) 9:1347–8. 27388821 10.1016/j.jcin.2016.04.030

[129] Kleber FX Rittger H Ludwig J Schulz A Mathey DG Boxberger M Drug eluting balloons as stand alone procedure for coronary bifurcational lesions: results of the randomized multicenter PEPCAD-BIF trial. *Clin Res Cardiol.* (2016) 105:613–21. 10.1007/s00392-015-0957-6 26768146

[130] Tsetis D Morgan R Belli A-M. Cutting balloons for the treatment of vascular stenoses. *Eur Radiol.* (2006) 16:1675–83. 16609863 10.1007/s00330-006-0181-x

[131] Vo MN Brilakis ES Grantham JA. Novel use of cutting balloon to treat subintimal hematomas during chronic total occlusion interventions. *Catheter Cardiovasc Interv.* (2018) 91:53–6. 10.1002/ccd.27184 29068125

[132] Venuti G D’Agosta G Tamburino C La Manna A. Coronary lithotripsy for failed rotational atherectomy, cutting balloon, scoring balloon, and ultra-high-pressure non-compliant balloon. *Catheter Cardiovasc Interv.* (2019) 94:E111–5. 10.1002/ccd.28287 31020765

[133] Shishehbor MH Zeller T Werner M Brodmann M Parise H Holden A A randomized trial of chocolate touch compared with lutonix drug-coated balloon in femoropopliteal lesions (The CHOCOLATE TOUCH Study). *Circulation.* (2022) 145:1645–54. 10.1161/CIRCULATIONAHA.122.059646 35377157

[134] Gemeinhardt O Schnorr B Speck U Scheller B. A novel paclitaxel coated balloon with increased drug transfer for treatment of complex vascular lesions. *PLoS One.* (2021) 16:e0259106. 10.1371/journal.pone.0259106 34714843 PMC8555778

[135] Cortese B Granada JF Scheller B Schneider PA Tepe G Scheinert D Drug-coated balloon treatment for lower extremity vascular disease intervention: an international positioning document. *Eur Heart J.* (2016) 37:1096–103. 10.1093/eurheartj/ehv204 26009594

[136] Bienek S Kusmierczuk M Mittag A Bettink S Scheller B. Novel, vessel anatomy adjusting drug-coated balloon-preclinical evaluation in peripheral porcine arteries. *Catheter Cardiovasc Interv.* (2020) 95:319–28. 10.1002/ccd.28592 31696642

[137] Speck U Häckel A Schellenberger E Kamann S Löchel M Clever YP Drug distribution and basic pharmacology of paclitaxel/resveratrol-coated balloon catheters. *Cardiovasc Intervent Radiol.* (2018) 41:1599–610. 10.1007/s00270-018-2018-9 29968090 PMC6132862

[138] Kong J Hou J Ma L Xing L Jia H Liu H Cutting balloon combined with paclitaxel-eluting balloon for treatment of in-stent restenosis. *Arch Cardiovasc Dis.* (2013) 106:79–85. 10.1016/j.acvd.2012.10.004 23527911

[139] Cortese B Micheli A Picchi A Coppolaro A Bandinelli L Severi S Paclitaxel-coated balloon versus drug-eluting stent during PCI of small coronary vessels, a prospective randomised clinical trial. The PICCOLETO study. *Heart.* (2010) 96:1291–6. 10.1136/hrt.2010.195057 20659948

[140] Cortese B Di Palma G Guimaraes MG Piraino D Orrego PS Buccheri D Drug-coated balloon versus drug-eluting stent for small coronary vessel disease: PICCOLETO II randomized clinical trial. *JACC Cardiovasc Interv.* (2020) 13:2840–9. 10.1016/j.jcin.2020.08.035 33248978

[141] Gobić D Tomulić V Lulić D Židan D Brusich S Jakljević T Drug-coated balloon versus drug-eluting stent in primary percutaneous coronary intervention: a feasibility study. *Am J Med Sci.* (2017) 354:553–60. 10.1016/j.amjms.2017.07.005 29208251

